# Targeting the nucleotide metabolism of *Trypanosoma brucei* and other trypanosomatids

**DOI:** 10.1093/femsre/fuad020

**Published:** 2023-05-08

**Authors:** Anders Hofer

**Affiliations:** Department of Medical Biochemistry and Biophysics, Umeå University, SE-901 87 Umeå, Sweden

**Keywords:** Trypanosoma, Leishmania, parasite, nucleotide metabolism, purine, pyrimidine, trypanosomiasis

## Abstract

African sleeping sickness, Chagas disease, and leishmaniasis are life-threatening diseases that together affect millions of people around the world and are caused by different members of the protozoan family *Trypanosomatidae*. The most studied member of the family is *Trypanosoma brucei*, which is spread by tsetse flies and causes African sleeping sickness. Nucleotide metabolism in *T. brucei* and other trypanosomatids is significantly different from that of mammals and was recognized as a target for chemotherapy already in the 1970–1980s. A more thorough investigation of the nucleotide metabolism in recent years has paved the way for identifying nucleoside analogues that can cure *T. brucei* brain infections in animal models. Specific features of *T. brucei* nucleotide metabolism include the lack of *de novo* purine biosynthesis, the presence of very efficient purine transporters, the lack of salvage pathways for CTP synthesis, unique enzyme localizations, and a recently discovered novel pathway for dTTP synthesis. This review describes the nucleotide metabolism of *T. brucei*, highlights differences and similarities to other trypanosomatids, and discusses how to exploit the parasite-specific features for drug development.

## Introduction

African sleeping sickness, or Human African Trypanosomiasis, is a fatal disease that is spread by tsetse flies in sub-Saharan Africa (Büscher et al. 2017). The human disease is caused by two subspecies of the protozoan parasite *Trypanosoma brucei. Trypanosoma brucei gambiense* causes chronic infections prevalent in western and central Africa and accounts for 97% of the cases, whereas the less common *Trypanosoma brucei rhodesiense*is responsible for an acute form of the disease in eastern Africa. When an infected fly bites, it injects parasites into the blood, where they multiply and cause the undulating fever that characterizes the first stage of the disease. The symptoms can easily be mistaken for other diseases, and it is often not until the second stage when the parasites infect the central nervous system that a proper diagnosis is made. This stage is characterized by a wide range of symptoms, including motor system and sensory disturbances, altered diurnal rhythm, personality changes, and in the end a fatal comatose condition. The progression of sleeping sickness is very dependent on the subspecies, and the chronic form caused by *T. b. gambiense* takes on average 3 years to become fatal, whereas *T. b. rhodesiense* kills the patient within weeks or months. Historically, the prevalence of the disease has been dependent on the political situation in Africa with major increases in times of war and political unrest. The situation was particularly urgent at the turn of the century with 25 000–30 000 reported yearly cases, which corresponds to 300 000–500 000 total cases based on estimations of the reporting frequency and the average duration of the disease. The situation has since then become much better with a >95% decrease in the yearly number of reported cases (Franco et al. 2020). However, 51 million people live in areas where there is a risk of becoming infected (Franco et al. 2020), and with a growing number of conflicts there is a risk that the number of cases will increase again. Key to disease control is proper surveillance and good treatment options to avoid vicious cycles where tsetse flies pick up the parasite from infected persons and spread it further.

*Trypanosoma brucei* belongs to a phylogenetic clade referred to as salivarian trypanosomes. In addition to the two human pathogenic *T. brucei* subspecies, this clade also includes *T. b. brucei, T. b. evansi, T. vivax*, and *T. congolense*, which cause anemia in a wide range of animals including cattle, horses, and camels (Kasozi et al. 2022). Most of the salivarian trypanosomes are spread by tsetse flies, which confines them to Africa. However, there are a few exceptions, including *T. b. evansi* and a South American subpopulation of *T. vivax*, which are spread mechanically by other flies without having life cycle stages in the insects. Another example is *T. b. equiperdum*, which causes a sexually transmitted venereal disease in horses. All salivarian trypanosomes are extracellular, and no major differences in their nucleotide metabolism have been reported except for some of the properties of their nucleoside/nucleobase transporters (described in the transporter section). However, it should be remembered that research on *T. congolense* and *T. vivax* is much more limited than on *T. brucei* and that there could be differences that have not yet been discovered. Although animal trypanosomiasis poses a high economic burden and millions of cattle and other domestic animals die every year because of it, all veterinary drugs in use against the disease were developed more than 30 years ago and are limited by side effects and drug resistance (Giordani et al. 2016, Kasozi et al. 2022). This is in stark contrast to the human disease where major improvements have been made in treatments as well as in the number of cases.

African sleeping sickness is not the only human disease caused by trypanosomes. In South and Central America, triatomine bugs spread *T. cruzi*, a non-salivarian trypanosome that causes Chagas disease in humans (Pérez-Molina and Molina 2018). The acute stage of the disease is generally mild but can occasionally give severe symptoms (1%–5% of the cases), and it often reappears as a chronic condition 10–30 years later. Approximately 6 million people are estimated to be infected with *T. cruzi*, and 30%–40% of the chronic cases suffer from cardiac, digestive, and/or neurological alterations that can sometimes be lethal. Acute cases can be successfully treated with benznidazole or nifurtimox, whereas treatment of the chronic cases fails in most cases. Another important disease caused by trypanosome-related organisms is leishmaniasis, which is spread by sandflies and is caused by several species of *Leishmania* (Burza et al. 2018). The yearly incidence is 0.7–1 million cases, with 50 000–90 000 being the visceral form of the disease that is nearly always fatal if not treated. In addition, the most common variant, the self-healing cutaneous form, can cause problems with remaining scars after the disease is cured, and the related mucocutaneous form can lead to severely disfiguring facial lesions. Current treatment for leishmaniasis depends on the symptoms as well as which species is causing it. Adverse effects and drug resistance are common problems.

*Trypanosoma* and *Leishmania* belong to the *Trypanosomatidae* family of parasites, and because *T. brucei* is comparatively easy to manipulate genetically, it has served as a model organism for the family. This review will therefore focus primarily on the nucleotide metabolism of *T. brucei*, but will also discuss the differences and similarities with *T. cruzi* and *Leishmania*, which affect more people. A major difference between the three trypanosomatids is that *T. brucei* is extracellular, whereas the main replicative form of *T. cruzi* and *Leishmania* in the mammalian host is the intracellular amastigote form. *T. cruzi* and *Leishmania* are taken up by the mammalian host cell into a parasitophorous vacuole, but *T. cruzi* needs to escape into the cytosol to start proliferation.

Nucleotide metabolism has made headlines on many occasions in the research history of *T. brucei* and other trypanosomatids. Such discoveries include the nucleobase analogue allopurinol, which is still one of the most commonly used drugs against canine and feline leishmaniasis as well as the discovery of that downregulation of the *T. brucei* P2 adenosine transporter is a common way to achieve resistance against two of the main classes of drugs used against African trypanosomiasis. In recent years, a much greater knowledge about nucleotide metabolism in these parasites has been acquired. In most cells, including our own, nucleotide metabolism is a mainly cytosolic process, whereas studies of the trypanosomatids have revealed an extensive interplay between different organelles in the process, including a new mitochondrion-dependent pathway for dTTP synthesis not observed in other organisms. The knowledge about trypanosomatid nucleotide metabolism has also led to advances in drug development such as orally available adenosine analogues that can cure *T. brucei*-infected animal models with brain infections. To cure *T. brucei* central nervous system infections by orally available drugs has been a holy grail of *T. brucei* research for decades. Despite major advances, the few reviews that exist from the last fifteen years focus only on specific parts of trypanosomatid nucleotide metabolism. A major incentive behind this proposal is to fill in this obvious lack of reviews by giving a broad and comprehensive picture of nucleotide metabolism in the trypanosomatids, to identify key drug targets, and to inspire new research in the area.

## *Trypanosoma brucei* life cycle stages and current treatment of African sleeping sickness

Vaccine development against African sleeping sickness has been hampered by the ability of *T. brucei* to switch its coat of variable surface glycoprotein, and we are completely dependent on medicines rather than vaccines to combat the disease (Büscher et al. 2017). *Trypanosoma brucei* is an extracellular parasite with several life cycle stages in the mammalian and tsetse fly hosts, and the target of chemotherapy is generally the long slender bloodstream form (BSF), which is the proliferating variant of the parasite in the mammalian blood, lymph, and central nervous system. It is enough to stop the proliferation of the parasites to cure the disease because in the absence of cell division, the variable surface glycoprotein switching process cannot occur and the parasites are easily taken care of by the immune defense. A certain fraction of the long slender BSFs develop into nondividing short stumpy BSFs adapted for transmission to tsetse flies. Inside the tsetse fly's midgut, the ingested parasites transform into the procylic life cycle stage that is able to proliferate again. Most of the studies on *T. brucei* have been performed on the long slender BSFs, which are most relevant for the disease, and procyclics.

The treatment of African sleeping sickness varies depending on both the disease stage and the subspecies causing it (Kasozi et al. 2022) as shown in Table 1. The first stage of the disease can be treated with pentamidine for *T. b. gambiense* and with suramin for *T. b. rhodesiense*, whereas a challenge has been that central nervous system infections can only be treated with drugs that can efficiently cross the blood-brain barrier. The treatment of second-stage *T. b. gambiense* infections has gradually improved, with the standard treatment being a nifurtimox-eflornithine combination therapy (NECT). The eflornithine component, which is also called difluoromethylornithine (DFMO), was initially used as a single treatment. The subsequent inclusion of nifurtimox made it possible to reduce the treatment period significantly, although the treatment is still quite demanding with slow infusions of 400 mg of DFMO every 12 hours for 7 days (the nifurtimox component is given orally). In recent years, the orally available drug fexinidazole has therefore become an attractive alternative against *T. b. gambiense* infections in remote areas with limited hospital access (Kasozi et al. 2022). The medicine can be used against both stages of the disease, but NECT is still recommended for advanced central nervous system infections. Second stage *T. b. rhodesiense* infections are even more difficult to treat than *T. b. gambiense*, and the treatment is based on melarsoprol, an arsenical that in 5%–18% of the cases leads to a life-threatening encephalopathy (Büscher et al. 2017). A phase II/III clinical trial performed by the Drugs for Neglected Disease initiative (HAT-r-ACC study) is currently evaluating the effect of fexinidazole on *T. b. rhodesiense* infections (Lee et al. 2020, Alvarez-Rodriguez et al. 2022).

**Table 1.** Current treatments of African sleeping sickness. The treatments are different depending on disease stage and subspecies. Abbreviations: Tbg, *T. b. gambiense*, Tbr, *T. b. rhodesiense*.

Treatment	Subspecies	2^nd^ stage effectiveness	Side effects	Application	Transporter
Pentamidine	Tbg	-	+	i.m.	AQP2 (and P2)
Suramin	Tbr	-	+	i.v.	
Melarsoprol	Tbr (Tbg)*	+	++	i.v.	AQP2 (and P2)
DFMO + nifurtimox	Tbg	+	+	i.v. (compl.)**	AAT6
Fexinidazole	Tbg	(+)	+	oral	

* Melarsoprol is no longer a common treatment option against *T. b. gambiense*.

** Complicated treatment due to large infusion volumes of DFMO.

Drug resistance has been a major problem for the treatment of both human and animal trypanosomiases, especially resistance against diamidines (e.g. pentamidine) and melaminophenyl arsenicals (e.g. melarsoprol). Interestingly, the first drug resistance gene discovered in *T. brucei* was *Tb*AT1, which encodes the purine transporter P2 and thereby makes a connection to nucleotide metabolism (Carter and Fairlamb 1993, Carter et al. 1995, Mäser et al. 1999). The natural substrates of this transporter are adenine and adenosine, but it was also found to be involved in the uptake of diamidines and melaminophenyl arsenicals. This transporter is particularly important for the uptake of diminazene aceturate (de Koning et al. 2004), a diamidine used as a veterinary drug, whereas resistance to pentamidine and melarsoprol was later found to be primarily mediated by mutations in an aquaporin named AQP2 (Baker et al. 2012, Graf et al. 2013, Munday et al. 2015). Drug resistance against the components in NECT (DFMO + nifurtimox) or against fexinidazole has not yet become a problem, but laboratory experience has shown that selection for resistance to nifurtimox or fexinidazole gives cross-resistance to both of them (Sokolova et al. 2010) and that drug resistance to DFMO can occur by losing the amino acid transporter AAT6 (Vincent et al. 2010). It is therefore important to develop more treatment alternatives, and the many differences in the nucleotide metabolism of *T. brucei* and other trypanosomatids as compared to humans can be exploited for the development of drugs with minimal side effects on the host cells.

## General nucleotide metabolism

**Figure 1.**
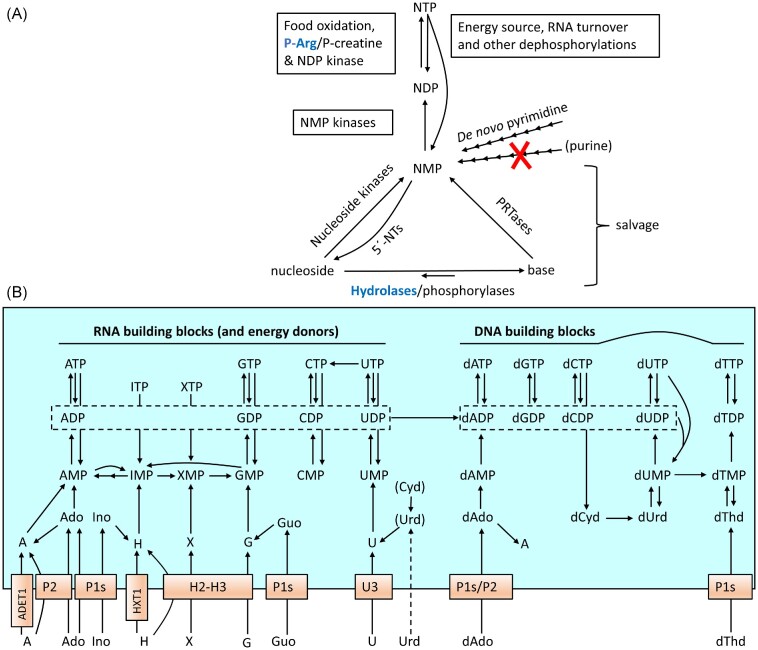
Overview of the nucleotide metabolism in *T. brucei*. (A) Schematic presentation of the intracellular nucleotide metabolism in trypanosomatids. The red cross indicates the lack of *de novo* purine biosynthesis and other differences compared to mammalian cells are marked in blue (followed by the mammalian alternative in black). (B) Expanded view of nucleotide biosynthesis in *T. brucei* long slender BSFs. The two boxes in the middle of Fig. 1B indicate that all four NDPs can be converted to the corresponding dNDPs, and the P1 transporters are in plural (P1s) because they represent an entire family of similar transporters. Minor uptake and phosphorylation/dephosphorylation activities are excluded, whereas Urd and Cyd are in brackets because their significance is unclear (only minor Urd uptake takes place in BSFs). Abbreviations: PRTases, phosphoribosyltransferases; NMP/NDP/NTP, nucleoside mono-, di-, or triphosphate; P-Arg, phosphoarginine; P-creatine, phosphocreatine; X, xanthine; H, hypoxanthine (one letter abbreviations are used for all nucleobases).

Food oxidation and general energy metabolism are central for the phosphorylation status of cellular nucleotides, with ATP driving the phosphorylation of the other nucleotides. Figure 1A shows a simplified nucleotide metabolism scheme with deviations in trypanosomatids compared to mammalian cells marked in blue. ATP and other nucleoside triphosphates (NTPs) are dephosphorylated and rephosphorylated in a recycling process where the same nucleotides can be used over and over again. In contrast, the supply of new nucleotides via the *de novo* and salvage synthesis pathways is primarily needed for the expansion of the total nucleotide pool during cell division or to replace degraded nucleotides. The most striking difference between trypanosomatids and mammalian cells indicated in Fig. 1A is the lack of *de novo* purine biosynthesis in the parasites (Ogbunude and Ikediobi 1983). This makes them dependent on salvaging preformed purine nucleosides and bases from the host to make new nucleoside monophosphates (NMPs). Nucleosides can either be salvaged directly or first cleaved to liberate nucleobases that are used instead. The cleavage can be performed by nucleoside hydrolases, which are dominant in trypanosomatids but not present in mammalian cells, or by phosphorylases. The trypanosomatids have a full set of enzymes to interconvert purine NMPs between each other, which allows them to survive with only one purine source. Hypoxanthine is generally the purine source in *T. brucei* growth media.

It is only *de novo* purine biosynthesis that is lacking in the trypanosomatids, whereas pyrimidines as well as deoxyribonucleotides (including dTTP) can be produced by *de novo* biosynthesis. The lack of *de novo* purine biosynthesis is very common among protozoan parasites and can be exploited by chemotherapy as described in the following sections. Figure 1B shows an expanded view of the nucleotide metabolism of *T. brucei* long slender BSFs. *Trypanosoma brucei* long slender BSFs and procyclics differ in which transporters they express, but other aspects shown in the figure are similar in the two life cycle stages. Dephosphorylation and phosphorylation reactions of minor importance are not included in this summarizing figure but will still be described in subsequent sections. Most of the reactions in the left part of Fig. 1B are also conserved in other trypanosomatids except for that the selectivities of the transporters are different and that *Leishmania* has the ability to deaminate adenine into hypoxanthine and thereby channel the majority of its purine salvage via hypoxanthine. However, it should be remembered that the nucleotide metabolism of other trypanosomatids have not been as extensively studied as *T. brucei*, particularly the deoxyribonucleotide metabolism shown to the right.

Figure 1B also includes the reactions catalyzed by ITP pyrophosphatase, which is a housekeeping enzyme that converts ITP (or dITP) and XTP to the corresponding monophospates (Vidal et al. 2022). ITP and XTP are deamination products of ATP and GTP, respectively, but can also be produced by the phosphorylation of the IMP and XMP intermediates in *de novo* purine biosynthesis. The purpose of the reaction is to prevent ITP/dITP and XTP from being incorporated into nucleic acids. Knockout studies in *T. brucei* showed that depleting the enzyme is not lethal by itself but sensitizes the trypanosomes to inhibitors of IMP dehydrogenase, most likely due to the buildup of IMP and consequently ITP (Vidal et al. 2022).

## Generation of NTPs from NDPs and NMPs

Glycosomes play a central role in ATP generation in *T. brucei* and other trypanosomatids (Pereira et al. 2011). These peroxisome-related organelles contain the enzymes needed for glycolysis, nucleotide sugar metabolism, the pentose phosphate shunt, and many of the enzymes required for nucleotide metabolism (Guther et al. 2014). The glycosomes have a particularly central role in *T. brucei* BSFs, which primarily live on carbohydrates and repress many of the mitochondrial reactions. In the insect midgut, there are limited amounts of carbohydrates available, and the procyclic form of the parasite expands the size of its single mitochondrion to allow the use of amino acids such as proline and threonine as energy sources (Pereira et al. 2011). In addition, ATP can also be generated from phosphoarginine in *T. brucei* and other trypanosomatids (Pereira et al. 2011) (Fig. 1A). This reaction is catalyzed by arginine kinase and has a similar role as creatine kinase in mammalian cells in rapidly generating ATP when the ATP generated from food oxidation is not sufficient. Although ATP is the main phosphorylation source in the cell, other NTPs participate as energy donors in dedicated reactions, including the usage of CTP in phospholipid metabolism, UTP in carbohydrate metabolism, and GTP in diverse processes such as protein translation and G-protein regulation. When used as energy sources, the NTPs themselves will be converted to NDPs and in a few processes to NMPs (e.g. in RNA turnover and phospholipid metabolism). In addition, NTPs, NDPs, and NMPs can also be dephosphorylated by side reactions of nonspecific phosphatases, but it is is unclear if this makes a significant contribution to the overall cellular nucleotide metabolism. The re-phosphorylation of NDPs and NMPs is catalyzed by NDP kinase and several types of NMP kinases, respectively (Fig. 1A).

Energy metabolism is very efficient in most organisms, and a rule of thumb is that the ATP:ADP:AMP ratio is 100:10:1 (Hardie 2018). The high cellular activity of NDP kinase and the NMP kinases ensures that the ratio of NTPs over NDPs and NMPs favors the triphosphate form for other nucleotides as well. In *T. brucei*, nucleotide pool measurements have mainly focused on NTPs, dNTPs, and ADP. The ATP pools are generally more than ten times higher than ADP but are very dependent on the supply of nutrients, and the ATP:ADP ratio can decrease within minutes upon growth medium removal in cell washing steps (Ranjbarian et al. 2022). It is therefore important to work fast and avoid washes when extracting nucleotides from *T. brucei* for analysis.

Despite their central role in nucleotide metabolism, knowledge about the NMP kinases and NDP kinase in trypansomatids is quite limited (Pereira et al. 2011). In mammalian cells, the NMP kinases include adenylate kinases for the phosphorylation of AMP/dAMP, guanylate kinases for the phosphorylation of GMP/dGMP, UMP-CMP kinases for the phosphorylation of UMP and CMP/dCMP and thymidylate kinase for dTMP (Panayiotou et al. 2014). However, the eukaryotic UMP-CMP kinases are not phylogenetically coherent. The majority of them are cytosolic and belong to a specific clade within the family of adenylate kinases but there is also a mitochondrial isoenzyme that is more related to thymidylate kinases (Fukami-Kobayashi et al. 1996, Xu et al. 2008). Generally, NDP kinase and most of the NMP kinases can phosphorylate ribonucleotides as well as deoxyribonucleotides, and dTMP is the only deoxyribonucleotide that needs a specific enzyme for its phosphorylation (thymidylate kinase). One factor that complicates the studies of the kinases in *T. brucei* is the many isoenzymes, including the following:

- six classical adenylate kinases (ADK:A-F)
- one UMP/CMP kinase with additional AMP specificity (ADKG)
- one nuclear adenylate kinase (ADKn)
- four NDP kinases (NDPK1-4)
- two guanylate kinases
- two thymidylate kinases

The large number of adenylate kinases in *T. brucei* and other trypanosomatids is unique among unicellular organisms, which generally is in the range from 1 to 3 (Ginger et al. 2005), whereas humans and other multicellular organisms have even more members than the trypanosomatids (Panayiotou et al. 2014). Studies of the *T. brucei* adenylate kinases show that the isoenzymes differ in localization with one of them in the glycosome (ADKD), three of them associated with the flagellum, and the remaining ones of uncertain location but thought to be in the mitchondrion and cytoplasm (Ginger et al. 2005). One of the studied enzymes (*T. brucei* ADKG) was phylogenetically categorized as a cytosolic CMP-UMP kinase but contains an amino acid substitution that allows it to recognize AMP (Ginger et al. 2005). The substrate specificity in descending order is CMP > AMP > UMP (Ginger et al. 2005), and the high affinity for CMP (∼20 times lower K_m_ than for AMP) could be an adaptation to the fact that *T. brucei* has unusually low cytidine nucleotide pools (Hofer et al. 2001). Subsequently, it was found that the trypanosomatids also have an adenylate kinase of the nuclear class (ADKn), and experiments in *T. cruzi* showed that the enzyme shuttles between the nucleus and the cytoplasm (de los Milagros Cámara et al. 2013). Most of the adenylate kinases are conserved in the trypanosomatids, and all *T. brucei* members except one of the flagellar isoenzymes have homologues in *T. cruzi* as well (Bouvier et al. 2006).

The NDP kinases are also compartmentalized, and studies from *T. cruzi* show specific isoenzymes for the flagellum/microtubules (NDPK2), glycosome (NDPK3), and cytosol/nucleus (NDPK1) (Miranda et al. 2022). Surprisingly, NDPK1 can also be excreted and it has been proposed that its extracellular activity might have an immunoregulatory role important for virulence (Miranda et al. 2022). From a drug development perspective, it is unclear if it is enough to inhibit specific NDP kinases or NMP kinases in order to kill *T. brucei* or other trypansomatids. RNAi studies on the adenylate kinases in *T. brucei* show that reduced expression of some of them can at least reduce cellular growth (Ginger et al. 2005). The properties of the NMP kinases and NDP kinases are important to consider for drug development because they are central for the activation of nucleoside analogues, which in most of the studied cases need to be converted to the triphosphate form in order to have any effect. However, it should be pointed out that many of the recently discovered analogues were identified by phenotypic screening and that mechanism of action studies are therefore often lacking. For natural nucleosides, it is generally the first phosphorylation step from the free nucleoside to the monophospate that is rate-limiting (Arnér and Eriksson 1995), but this is not always the case for nucleoside analogues. It would therefore be of value to know more about the specificities of the NMP kinases and NDP kinases for future nucleoside analogue development.

## Nucleoside and nucleobase transport

It is essential for *T. brucei* and other trypanosomatids to be able to efficiently take up purines from the environment. Most of the uptake is performed by a family of equilibrative nucleoside transporters (ENTs), which is the dominating nucleoside transporter family also in mammalian cells, but the ones studied in trypanosomatids are not equilbrative and are instead driven by the H^+^ gradient (Campagnaro and de Koning 2020). The H^+^ gradient dependency has been verified for the *T. brucei* P1, H1, and H2 transporters and may be general for the trypanosomatid ENT transporters (de Koning and Jarvis 1997a, de Koning and Jarvis 1997b, de Koning et al. 1998). This active transport system can be exploited in drug discovery by using nucleoside/nucleobase analogues that are preferentially taken up by the parasites compared to mammalian cells. The trypanosomatid transporters are generally specific for either nucleosides or nucleobases, although there are some exceptions that transport both (Table 2). Most of the transporters listed for *Leishmania mexicana* have also been verified in *Leishmania major* and *Leishmania donovani*, indicating that they seem to be conserved in *Leishmania* (Alzahrani et al. 2017). In *T. brucei*, there is a fairly strict division between purine and pyridimidine specificities, whereas some of the *T. cruzi* and *Leishmania* nucleoside transporters have more relaxed specificities (especially *Lm*UU1). Some other general conclusions are that cytidine transport is limited in all three trypanosomatids shown in Table 2 and that adenosine transport is much more efficient in *T. brucei* and *Leishmania* than in *T. cruzi*. In comparison, mammals have broad-range ENTs with only limited ability to discriminate between different nucleosides, and they often recognize some nucleobases as well (Wright and Lee 2021). In addition, mammalian cells have a few nucleobase transporters (Inoue 2017) and a separate class of sodium-dependent concentrative nucleoside transporters, but these generally have a more narrow tissue distribution (Wright and Lee 2021).

**Table 2.** Transporters in *Trypanosomatidae*. Substrates with affinities 30–300 times lower than than of the best substrate are in brackets (double brackets indicate even lower affinity). The table is based on the data from a review on transporters in pathogenic protozoans (Campagnaro and de Koning 2020) and includes mainly natural substrates. An exception is that the tubercidin recognition by *Tcr*NT2 is included in order to highlight that the transporter can bind purines. One-letter abbreviations are used for the nucleobases with H = hypoxanthine and X = xanthine.

Species	Transporter	Life cycle stage	Substrates	Comments
*T. brucei*	P1s (NT2-7,9–10)*	BSF/PCF	Ado/dAdo, Ino/dIno, Guo/dGuo (Thd)**	
	P2 (AT1)	BSF	A, Ado/dAdo	Drug resistance
	H1	PCF	G, H, X	
	H2	BSF	G, H (A, X, Guo)	High affinity
	H3	BSF	G, H, A, X	Low affinity
	H4 (NBT1/NT8.1)	PCF (BSF)	G, H, X, A (Guo/Ino)	
	NT11 and 12		*Unclear*	
*Not ENTs:*	U1	PCF	U (Urd)	
	U2	PCF	Urd	Minor transport
	U3	BSF	U ((Urd))	
	C1	PCF	C	Minor transport
	ADET1	BSF/PCF	A	
	HXT1	BSF	Hx	Minor transport
*L. mexicana*	NT1 (NT1.2)		Ado (Thd/Urd/Cyd)	
	UUT1 (NT1.1)		Urd, Ado (Ura)	
	NT2		Guo, Ino	
	NT3 (NBT1)		A, G, H, X	
	NT4		A (H)	Acid-activated
*Not ENT:*	U1		U (Urd)	
*T. cruzi*	NB1		H, G, (A/X)	
	NB2		*Not characterized*	Homology: *Lm*NT4
	NT1		Ino, Guo (Ado/Hx)	
	NT2		Thd, dUrd (Urd/Tubercidin)	
	Uracil?***			

* NT3 and NT4 are inactive.

** Indicated substrate specificities are for NT2. The ability to bind Thd (and H) varies among P1s.

*** Uracil is taken up, but the identity of the transporter is unknown.

The two adenosine transporter types in *T. brucei*, P1 and P2, have received the most attention (Table 2). Transporters from the P1 family have a general purine nucleoside specificity, and their main substrates are adenosine, inosine, and guanosine, as well as the corresponding deoxyribonucleosides (de Koning and Jarvis 1999). There are several P1 transporter genes (*Tb*NT2–7 and 9–10) distributed on different chromosomes, whereas the P2 transporter is encoded by the single *Tb*AT1 gene (note the different numbering of the protein and gene). Due to the multitude of P1 transporters, which are fairly similar to each other in their substrate specificities, it is considered more or less impossible to acquire resistance to drugs by downregulating this transport activity. The opposite is true for the P2 transporter, which is easily lost in drug-exposed parasites. The P2 transporter is only expressed in BSFs and recognizes a pattern of amino groups in the adenine base, and this pattern is lacking in other purines but is shared in diamidines and melaminophenyl arsenicals. The ribose moiety has only a modest influence on the interaction, and the P2 transporter is active with adenine as well as the corresponding nucleosides adenosine and deoxyadenosine. The recognition patterns of P1 and P2 will be further described in the drug development sections. Interestingly, *T. congolense* and *T. vivax* that cause animal trypanosomiasis lack P2 altogether and have only one major P1 transporting activity in each species (Ungogo et al. 2023). The single *T. congolense* P1 transporter is called *Tco*AT1 or *Tco*NT10 (not to be confused with *Tb*AT1 from *T. brucei*, which is a P2 transporter), and the only *T. vivax* P1 transporter that could be confirmed to be active is called *Tvx*NT3. Transport studies showed that both *Tco*AT1 and *Tvx*NT3 are purine-specific and recognize typical P1 substrates (adenosine, inosine, and guanosine), but *Tvx*NT3 has a broader substrate specificity and can also bind purine nucleobases (not tested as substrates). Similarly to the *T. brucei* P1 transporters (but unlike P2), *Tco*AT1 and *Tvx*NT3 does not seem to transport drugs tested from the diamidine and melanophenyl arsenical families (Munday et al. 2013, Ungogo et al. 2023). In addition to the purine nucleoside transporters, all three species have several genes encoding purine nucleobase transporters, although they have only been characterized in *T. brucei*. In *T. brucei* the expression pattern of H1-H4 varies with the life cycle stage with H2 and H3 being expressed in BSFs (Fig. 1B, Table 2). H2 is a high-affinity hypoxanthine/guanine transporter (with lower affinities for other substrates), whereas H3 has a ∼50-fold lower affinity than H2 and is likely to be of lower importance (de Koning and Jarvis 1997a, Wallace et al. 2002). In addition, there are transporters specific for adenine (ADET1) and hypoxanthine (HXT1) that were identified in BSFs when other transporters were knocked out, but they are most likely of minor importance in wild-type trypanosomes (Campagnaro et al. 2018).

Pyrimidines can be made by *de novo* synthesis in trypanosomatids, and the transporters, which are generally not of the ENT type, are therefore of less importance. *T. brucei* BSFs only have a single pyrimidine transporter (U3), which is highly specific for uracil (Ali et al. 2013a). Surprisingly, the transporter does not efficiently use uridine (it has 10 000 times lower affinity than uracil), even though this is the major pyrimidine source in the blood and cerebrospinal fluid (Eells and Spector 1983a). Thus uridine transport is shown in double brackets in Table 2. BSFs are also capable of low-affinity thymidine transport, but this activity is strongly inhibited by adenosine and inosine, which indicates that it is a side activity of the P1 transporter (Ali et al. 2013a). In procyclics, there is a larger set of pyrimidine transporters with U1 being specific for uracil (and to a lesser extent uridine), U2 being specific for uridine, and C1 being specific for cytosine (Gudin et al. 2006). However, the physiological function of the C1 transporter is unclear; its activity is low and the trypanosomes do not have the necessary enzymes to salvage cytosine. It can therefore be questioned if cytosine is the main substrate or if the transporter has any other unknown function.

In addition to the nucleoside/nucleobase transporters in the plasma membrane, there is also a need to take up nucleotides into different organelles. Most organelles need the nucleotides as energy sources, whereas glycosomes and the mitochondrion participate directly in nucleotide synthesis and therefore have the need to also take up nucleosides and/or nucleobases (Guther et al. 2014, Moro-Bulnes et al. 2019). Generally, the uptake process requires transporters, with the exception of the passage through the nuclear and outer mitochondrial membranes, which have pores large enough to let nucleotides pass freely. As described below, glycosomal nucleobase transport might also be an exception. Very little is known about organellar transport in the trypanosomatids, but from other eukaryotes it is known that a general difference compared to the plasma membrane is that many of the organelles have the ability to transport nucleotides, whereas the plasma membrane only takes up nucleosides and nucleobases. *T. brucei* has some nucleotide transporter homologues (Colasante et al. 2009), but it is only the mitochondrial ADP/ATP carrier (*T. brucei* MCP6) that has so far been confirmed to have a transporting activity (Peña-Diaz et al. 2012). In *T. brucei* procyclics, this transporter exchanges the ATP produced in the mitochondrial matrix for ADP needed as substrate in oxidative phosphorylation, whereas the transporter is operating in the reverse direction in BSFs that generate most of their ATP in the glycosomes instead. It is not known how the glycosomes take up nucleotides, but there is evidence that they similarly to peroxisomes contain pores for the exchange of small hydrophilic molecules with the cytosol (Antonenkov and Hiltunen 2012, Gualdron-López et al. 2012). These pores are likely to be responsible for the uptake of nucleobases into the glycosomes but not bulkier metabolites such as ATP. For comparison, mammalian peroxisomes have an ATP-AMP exchanger as well as transporters for coenzymes and other substances that cannot be taken up through the pores (Antonenkov and Hiltunen 2012).

Although extracellular nucleotides are not taken up directly by the plasma membrane, such uptake can still occur indirectly by using ectonucleotidases (ecto-NTs), which are present on the surface of both the trypanosomatids (Cosentino-Gomes and Meyer-Fernandes 2011) and the host cells (Giuliani et al. 2020). The ecto-NTs dephosphorylate the nucleotides into nucleosides that can be taken up. There are several types of ecto-NTs including NTP diphosphohydrolases, 5´-ecto-NTs and 3´-ecto NTs. NTP diphosphohydrolases and 5´-ecto-NTs dephosphorylate NTPs/NDPs and NMPs, respectively, whereas 3´-ecto-NTs can use 3´-nucleotides or nucleic acids as substrates. It is unclear how important the parasitic ecto-NTs are for nutrient uptake (Cosentino-Gomes and Meyer-Fernandes 2011). Nucleosides/nucleobases are more abundant than nucleotides in the extracellular environments in the mammalian host, whereas they have not been measured in the parasitophorous vacuole where *Leishmania* amastigotes reside. In contrast, the cytosol where *T. cruzi* amastigotes proliferate is heavily dominated by nucleotides that are present in millimolar concentrations (Traut 1994). However, it is also unclear in this case how important the *T. cruzi*´s own ecto-NTs are for this process because there are also cytosolic 5´-NTs in the host cells that can dephosphorylate NMPs and thereby provide nucleosides to the intracellular parasites.

Nucleotides/nucleosides are also important signalling molecules in plasma and other extracellular fluids, and it has been suggested that the main purpose of the 5´-ecto-NTs/NTP diphosphohydrolases of *T. brucei* and the extracellular life cycle stages of *T. cruzi* and *Leishmania* is to disrupt cell signalling in the host immune defense (Cosentino-Gomes and Meyer-Fernandes 2011). The 3´-ecto-NTs are primarily important for *Leishmania* among the human pathogenic trypanosomatids (Freitas-Mesquita and Meyer-Fernandes 2017). When the parasite has been taken up by its insect vector, the 3´-ecto-NTs might possibly have a role in providing purines from nucleic acids ingested by the sand fly, and in the mammalian host they have been suggested to help the parasite to escape the immune defense by degrading the nucleic acid component of neutrophil extracellular traps (Guimarães-Costa et al. 2014). These effects are dependent on the nuclease function of the enzyme, whereas it is unclear whether the 3´-nucleotidase activity serves any purpose.

## Purine salvage

**Figure 2.**
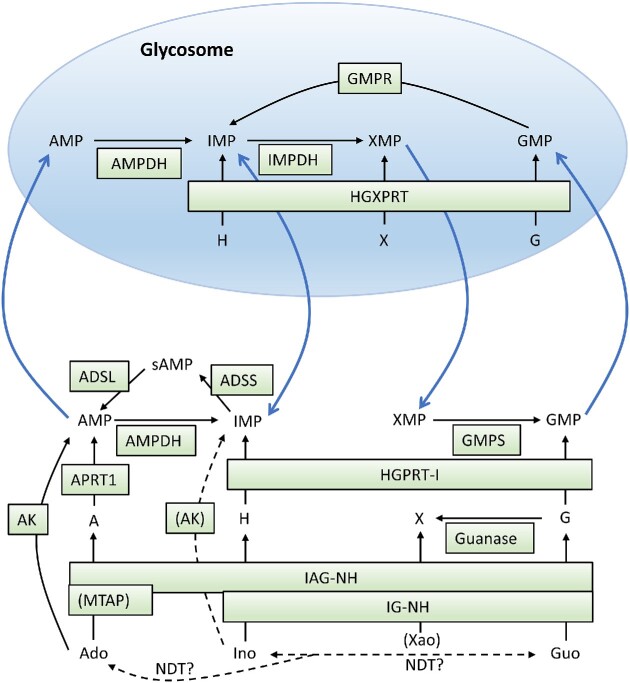
Cytosolic and glycosomal purine salvage and interconversion reactions in *T. brucei*. The blue arrows indicate necessary NMP movements over the glycosomal membrane in order to supply both compartments with AMP and GMP independently of purine source. Dashed lines indicate low enzyme activities. Abbreviations: IMPDH, IMP dehydrogenase; GMPR, GMP reductase; sAMP, succinyladenylate; AK, adenosine kinase; APRT1, adenine phosphoribosyltransferase 1 (cytosolic isoform); HGXPRT, hypoxanthine-guanine-xanthine phosphoribosyltransferase; IAG-NH, inosine-adenosine-guanosine-nucleoside hydrolase; IG-NH, inosine-guanosine-nucleoside hydrolase; ADSL, adenosylsuccinate lyase; ADSS, adenylosuccinate synthase; GMPS, GMP synthase; AMPDH, AMP dehydrogenase; HGPRT-I, hypoxanthine-guanine phosphoribosyltransferase 1 (cytosolic isoform); MTAP, methylthioadenosine phosphorylase; NDT, nucleoside deoxyribosyltransferase. Isoenzymes that are inactive or not expressed in BSFs are not included (glycosomal enzymes APRT2 and HGPRT-II, respectively).

*Trypanosoma brucei* and other trypanosomatid pathogens are able to use all purine nucleosides and bases known to be salvageable in nature and to interconvert the formed NMPs (Fig. 2). Interestingly, the cytosol can salvage all the nucleosides and bases and has a full set of enzymes to interconvert AMP and IMP, whereas some of the enzymes needed for the interconversion of IMP and GMP are only present in the glycosomes in *T. brucei* (Guther et al. 2014) (Fig. 2). It is not known if there is any benefit from this division of labor between the two compartments, but a consequence is that a trypanosome that lives on adenosine or hypoxanthine, which are two major purines in the blood, can make AMP (and IMP) in the cytosol, but in order to make cytosolic GMP it needs to transport IMP to the glycosome, convert it to XMP, and take it back to the cytosol to form GMP. A corresponding division of labor between the two compartments is also required to make AMP from GMP, where the first step occurs in the glycosome and the last two steps in the cytosol. The glycosomal membrane therefore needs to allow the passage of NMPs, but it is currently not known how it is mediated. Both compartments have their own adenylate kinases, guanylate kinases, and NDP kinases for the further phosphorylation of AMP and GMP to their triphosphate forms. A distinction between the glycosome and the cytosol is that the glycosome only has enzymes to salvage nucleobases, whereas the cytosol can also salvage nucleosides. Perhaps the small pores in the glycosomal membrane allow more efficient passage of nucleobases than the larger nucleosides.

Nucleosides can be salvaged in two different ways. In *T. brucei*, most nucleosides are first cleaved and the corresponding nucleobases are salvaged into NMPs (Parkin 1996), with the notable exception of adenosine that can also be directly phosphorylated to form AMP (Lüscher et al. 2007, Vodnala et al. 2008). Adenosine is the purine source that has received the most attention in *T. brucei*, both because adenosine analogues have shown good promise against the disease and because the P2 adenosine transporter is involved in multidrug resistance. Adenosine can be salvaged in two different ways. The first way is a two-step procedure to cleave the substrate via the enzyme inosine-adenosine-guanosine nucleoside hydrolase (IAG-NH) and then to salvage the formed adenine (Parkin 1996). The second way to salvage adenosine is to phosphorylate it directly with adenosine kinase. This enzyme has a more than 100-fold higher adenosine affinity (and catalytic efficiency) than IAG-NH (Parkin 1996, Vodnala et al. 2008), which makes it likely to be the major salvage route under conditions when the substrate concentration is low.

The combination of having highly efficient transport and phosphorylation activities is an advantage for developing adenosine analogues that are specifically salvaged by the parasite. For comparison, mammalian cells also have a high-affinity adenosine kinase (Sahin et al. 2004) but less efficient adenosine transport (Campagnaro and de Koning 2020, Wright and Lee 2021). There are two isoforms of adenosine kinase in the parasite, but they are nearly identical in amino acid sequence and are considered to be functionally equivalent (Vodnala et al. 2008). *T. brucei* adenosine kinase can phosphorylate adenosine, deoxyadenosine, and inosine, but the high affinity for adenosine (K_m_ = 0.041 µM) makes it the preferred substrate (Vodnala et al. 2008). In contrast, the enzyme activity with inosine is much lower and is marked in brackets in Fig. 2 (see the drug development section for enzyme kinetic data). Similarly to the mammalian enzyme, the *T. brucei* adenosine kinase is subject to substrate inhibition by adenosine in the micromolar range (Vodnala et al. 2008).

The extreme efficiency of adenosine salvage may also explain the symptoms of late-stage sleeping sickness. Experiments in mice with *T. brucei* brain infections showed a disruption of the host adenosine signalling system known to be important for sleep regulation, and this might explain why the disease leads to dysregulated diurnal rhythm (Rijo-Ferreira et al. 2020). The adenosine concentration is normally only 0.12 µM in the cerebrospinal fluid of rodents (Eells and Spector 1983b) and is below the detection limit of 0.1 µM in humans (Eells and Spector 1983a). With the high affinity of adenosine kinase, it would not be surprising if the parasites can decrease the concentration further and disrupt the associated signalling pathways, although more evidence is required to support this hypothesis.

A comparison of purine salvage between *T. brucei* and *Leishmania* shows some differences. For example, *L. donovani* adenosine kinase is not subject to substrate inhibition, and it has a comparably low affinity for adenosine (K_m_ = 33 µM) (Datta et al. 1987). The major route of salvage seems instead to occur via cleavage to adenine, which is deaminated by adenine amidotransferase, a *Leishmania*-specific enzyme that does not exist in other studied trypanosomatids or in mammalian cells (Boitz and Ullman 2013). Although the parasite also has APRT and can use adenine directly, the primary pathway in *Leishmania* is to deaminate the substrate and salvage the hypoxanthine using XPRT (homologous to *T. brucei* HGXPRT) or HGPRT (Boitz and Ullman 2013).

Figure 2 also includes reactions catalyzed by nucleoside deoxyribosyltransferase (NDT), an enzyme present in most trypanosomatids, which can interconvert deoxyribonucleosides by replacing the nucleobase attached to them with other nucleobases in the surroundings. Studies of *T. brucei* NDT, which is purine-specific, have shown that although it is primarily used for the interconversion of purine deoxyribonucleosides, it can also use ribonucleosides but with a 40-fold lower activity (Del Arco et al. 2019). The reactions are therefore indicated with question marks in the figure. Perhaps the low activity can be compensated for by the higher concentrations of the ribonucleosides compared to deoxyribonucleosides. Guanosine and inosine can freely interconvert between each other but the higher physiological concentration of hypoxanthine compared to guanine is likely to favor inosine formation. Inosine (or guanosine) will in turn drive adenosine formation. The unidirectional arrow between the inosine/guanosine equilibrium and adenosine indicates that the oxopurines are superior ribosyl donors compared to adenosine (25-fold difference).

Many of the enzymes in *T. brucei* purine salvage have been knocked out or knocked down, and the results support the expected roles of the different enzymes in purine salvage.

- HGPRT/HGXPRT. Knocking down both enzymes is required to give a strong effect on *T. brucei* proliferation with hypoxanthine as the purine source, whereas it is sufficient to knock down HGXPRT alone when xanthine is used (Doleželová et al. 2018).
- APRT. Knocking down APRT leads as expected to strongly reduced *T. brucei* proliferation when adenine is used as the purine source (Doleželová et al. 2021).
- Adenosine kinase. Knocking down the enzyme has no effect on *T. brucei* proliferation with hypoxanthine or supraphysiological concentrations of adenosine as the purine source (Lüscher et al. 2007). This is in line with the observation that the enzyme is only important for high-affinity adenosine salvage (Vodnala et al. 2008).
- IAG-NH/IG-NH/MTAP. Knocking down IAG-NH, IG-NH, or MTAP (methylthioadenosine phosphorylase) gives only a slight reduction in *T. brucei* proliferation using hypoxanthine-containing medium, but over time an increased number of abnormal cells accumulate when MTAP or IG-NH is knocked down (Berg et al. 2010). The role of MTAP in regular nucleotide metabolism is probably of minor importance, but by being able to cleave methylthioadenosine it has an important role in the methionine cycle and polyamine synthesis, which might explain the accumulation of abnormal cells when the enzyme is knocked down.

## Purine sources and inhibitors of purine salvage

Purine salvage has been seen as an obvious target for drug discovery against the trypanosomatids ever since the lack of *de novo* purine biosynthesis was discovered in the 1970–1980s (Marr et al. 1978, Gutteridge and Gaborak 1979, Fish et al. 1982). Subsequently, it has been shown that the plasma level of salvageable purines is a limiting factor for the proliferation of salivarian trypanosomes as shown in *T. musculi*-infected mice (Albright and Albright 1988). However, the major purine sources in human plasma available for salvage are a matter of controversy because the analyses are very sensitive to how the samples are handled. Purines are continuously excreted by cells and metabolized to uric acid, which is by far the most abundant purine in human plasma but is not possible to salvage. In order to quantify the plasma levels of salvageable purines, care must be taken to immediately inactivate metabolizing enzymes and quickly remove the cells from the blood when collecting the sample to avoid changes in the purine concentrations during handling. Most studies indicate that hypoxanthine and xanthine are the major salvageable purine sources, but when human blood samples were directly collected into a vial containing inhibitors of adenosine deaminase and xanthine oxidase followed by rapid filtration to remove the cells, adenosine was highest (2 µM) followed by hypoxanthine/xanthine (totally 0.7 µM) and inosine (0.2 µM) (Slowiaczek and Tattersall 1982). Hypoxanthine and xanthine were inseparable in the analysis and were therefore given a collective value. Guanosine and guanine are not detectable in most analyses and are probably of less importance. The natural purine concentrations also vary greatly over time depending on a wide range of factors such as physical exercise and diet, and it has been reported that the plasma hypoxanthine concentration can increase ∼20 fold after exercise (Kaya et al. 2006). The concentrations of salvageable purines have also been measured in cerebrospinal fluid, and in this case hypoxanthine and xanthine are the dominating purines, whereas adenosine is below the detection limit in humans (Eells and Spector 1983a). A general conclusion from all of these studies is that depending on the situation it will either be adenosine or hypoxanthine/xanthine that is the dominating purine source for *T. brucei*. For the intracellular trypanosomatids, the situation is even more unclear. The host cell cytoplasm is heavily dominated by nucleotides over nucleosides/nucleobases, and it is difficult to measure the latter reliably due to their fast metabolism and sensitivity to disturbances during harvesting. Nothing is known about purine concentrations in the parasitophorous vacuole where *Leishmania* amastigotes reside.

From a drug development perspective, there are two main ways to exploit the salvage pathways:

- Inhibiting (or hyperactivating) important enzymes for salvage and interconversion.
- Using substrate analogues of salvage enzymes that convert the analogues into their nucleotide forms and subsequently inhibit downstream processes.

Despite major efforts to inhibit the salvage enzymes, only limited progress has been made, and currently the strategy of using substrate analogues looks more promising. Generally, experiments on salvage enzyme inhibitors are not always straightforward to interpret. A complication is that the inhibition is very dependent on the purine source used in the growth medium, and it is therefore difficult to translate the *in vitro* inhibition constants to the situation in the mammalian host. Furthermore, the purine concentrations vary in different animal models as well as in different body fluids and tissues. Other aspects to be aware of with cultured trypanosomes are that they need an adaptation period when changing purine source in the culture and that there can be purines in the growth medium coming from the serum component. This multitude of complications can possibly explain why drug development projects on substrate analogues covered in the next section have been much more successful than inhibiting salvage. Nevertheless, there are a few interesting findings. Generally, the inhibitors can be divided into those that block early steps in salvage and those that block the interconversion between AMP, IMP, and GMP. Early step blockers include APRT inhibitors, dual HGPRT-HGXPRT inhibitors (Doleželová et al. 2018), and dual inhibitors of IAG-NH and IU-NH (Berg et al. 2010). Generally, the inhibitors conditionally inhibit trypanosome proliferation *in vitro* and the parasites can still live on other purine sources. The HGPRT-HGXPRT inhibitors have the broadest effect due to their ability to block the salvage of all oxypurines and thus force the trypanosomes to only live on adenosine/adenine as a purine source. However, *in vivo* data would be necessary to shed light into whether it is sufficient to block oxopurine salvage to cure *T. brucei* infections or if the natural levels of adenosine/adenine are sufficient to circumvent the inhibition. An alternative approach is instead to target the enzymes responsible for interconversion between AMP, IMP, and GMP. Especially GMP synthase might be an attractive target because its inhibition makes the trypanosomes dependent on the salvage of guanine/guanosine, which generally are undetectable in the blood and other body fluids. Accordingly, GMP synthase knockout *T. brucei* can only be cultured in media containing guanine and cannot establish infection in mice (Li et al. 2015). Another way to block the pathway from IMP to GMP is to inhibit IMP dehydrogenase. Ribavirin is a drug that inhibits *T. brucei* proliferation *in vitro* by blocking both IMP dehydrogenase and the related enzyme GMP reductase (Bessho et al. 2016). However, there is a possibility to circumvent the inhibition by the salvage of xanthine, and GMP synthase is therefore likely to be a better target.

An alternative option is to inhibit the two enzymes needed for AMP synthesis from IMP (ADSL and ADSS). Knockout studies in *Leishmania* showed that this made the parasites dependent on exogenous adenosine or adenine (Boitz et al. 2013), and experiments in *T. brucei* showed that reduced expression of either of the two enzymes slowed down proliferation both *in vitro* (with hypoxanthine as the purine source) and in *T. brucei*-infected mice (Mony et al. 2014). Interestingly, the experiments on *Leishmania* showed that the *in vivo* phenotype of the ADSS knockout was mild in comparison to the ADSL knockout, where the entrapment of the purines as succinyl-AMP was hypothesized to be the reason for the severely compromised ability to infect mice. The synthesis of succinyl-AMP is generally allosterically inhibited by adenine nucleotides (AMP/ADP/ATP), and if AMP cannot be formed the regulation fails and IMP is used up for succinyl-AMP synthesis. This leads to a general purine deficiency due to the fact that IMP is a substrate for both AMP and GMP synthesis.

A special twist in drug development against the salvage enzymes was the discovery of a trypanocidal compound acting as a hyperactivator of adenosine kinase by preventing substrate inhibition (Kuettel et al. 2009). Hyperactivators are commonly used to activate receptors (referred to as agonists), but this was, according to the authors, the first time ever that a metabolic enzyme was targeted by a hyperactivator.

## Substrate analogues of purine salvage enzymes

Substrate analogues of the salvage enzymes act as prodrugs that require cellular conversion by kinases or phosphoribosyltransferases (PRTases) into their active nucleotide forms. Most of the research focus has been on the first step when the monophosphate form is produced, which in the case of *T. brucei* purine synthesis can occur via HGPRT, HGXPRT, APRT, or adenosine kinase.

Pyrazolopyrimidines act as substrate analogues of purine PRTases (HGPRT-like enzymes and APRT). The pyrazolopyrimidines are, despite their name, purine analogues, and allopurinol is the one that has received the greatest attention as a drug against the trypanosomatids. This drug, which is widely used for the treatment of gout, proved to be effective against leishmaniasis and Chagas disease in humans already in the 1980–1990s and is still in use today, although less frequently for the human trypanosomatid-related diseases (Marsden et al. 1984, Gallerano et al. 1990, Mazzeti et al. 2019, Maguire et al. 2021, Madusanka et al. 2022). Instead, it has become a major treatment option against feline and canine leishmaniasis caused by *L. infantum* (Ribeiro et al. 2018, Garcia-Torres et al. 2022). Allopurinol resembles hypoxanthine, and its ribophosphorylation by HGPRT-like enzymes leads to the formation of the corresponding IMP analogue, which needs to be aminated and then further phosphorylated to the corresponding ATP analogue to block the RNA synthesis of the parasites. In *T. brucei*, allopurinol is a good substrate for HGPRT, but the amination of the formed monophosphate is not efficient (Fish et al. 1985). A much stronger antitrypanosomal effect can be achieved by aminopurinol, an aminated form of allopurinol that bypasses the amination step and is instead a substrate analogue of APRT (Natto et al. 2005), but the circumvention of the amination step also leads to greater toxicity in mammalian cells. This is because the selectivity of allopurinol is based on the fact that it is both a poor substrate of mammalian HGPRT (Keough et al. 1999) and is not aminated by mammalian cells (Spector et al. 1984). Aminopurinol is therefore not a good treatment option against *T. brucei* or other trypanosomatids. In contrast, allopurinol has very low toxicity and is therefore a much better option against the trypanosomatids that are sensitive to it.

In the case of *T. brucei*, substrate analogues of adenosine kinase have been much more successful than the nucleobases described above and were initially dominated by inosine analogues, including formycin B as well as 9-deazainosine (9-DINO) that cured *T. brucei*-infected mice, although with only limited effect against second-stage disease (Bacchi et al. 1987). Similarly to allopurinol, these analogues first form the corresponding IMP analogue and subsequently need to be aminated and further phosphorylated to the corresponding ATP analogue in order to have any effect on the trypanosomes. Formycin B monophosphate can also be aminated in mammalian cells and is therefore associated with side effects, whereas 9-DINO is more selective. Two advantages with inosine analogues compared to many adenosine analogues are that they do not need to be combined with adenosine deaminase inhibitors to be stable in the blood and that they are primarily taken up by the *T. brucei* P1 transporter family, which is not associated with drug resistance. Inosine was initially proposed to be phosphorylated by ATP-independent phosphotransferases (the previous term for 5´-nucleotidases), but later it was found that it can be phosphorylated by adenosine kinase (Vodnala et al. 2008). The k_cat_ with inosine is only ∼25% compared to adenosine, and the affinity is ∼10 000 times lower (K_m_ = 570 vs. 0.041 µM). This may look like a negligible activity, but the catalytic efficiency with this substrate is comparable to adenine arabinoside, an adenosine analogue that was proven to cure *T. brucei*-infected mice in an adenosine kinase-dependent manner, thus indicating that low-affinity substrates can also be used against the trypanosomes (Vodnala et al. 2008). It may seem like an enigma how the analogues have a chance to compete with adenosine despite 3–4 orders of magnitude lower affinity, but a possible explanation could be that adenosine entering the trypanosomes is metabolized so rapidly that it does not build up sufficient intracellular concentrations to act as a competitor. Although inosine analogues have shown success in acute mouse models, they have not been verified in mice with brain infections, and most of the development is instead focused on adenosine analogues to achieve a higher affinity for the enzyme. Nevertheless, there has been a renewed interest in inosine analogues in recent years due to the fact that they are taken up by the P1 transporter and thus are not associated with drug resistance. *In vitro* results indicate that some alkylated 7-deazainosine analogues inhibit *T. brucei* with EC_50_ values in the nM range (Hulpia et al. 2020a) and an evaluation of animal trypanosomiasis showed that one of the analogues could cure *T. congolense*-infected mice (Mabille et al. 2022).

**Figure 3.**
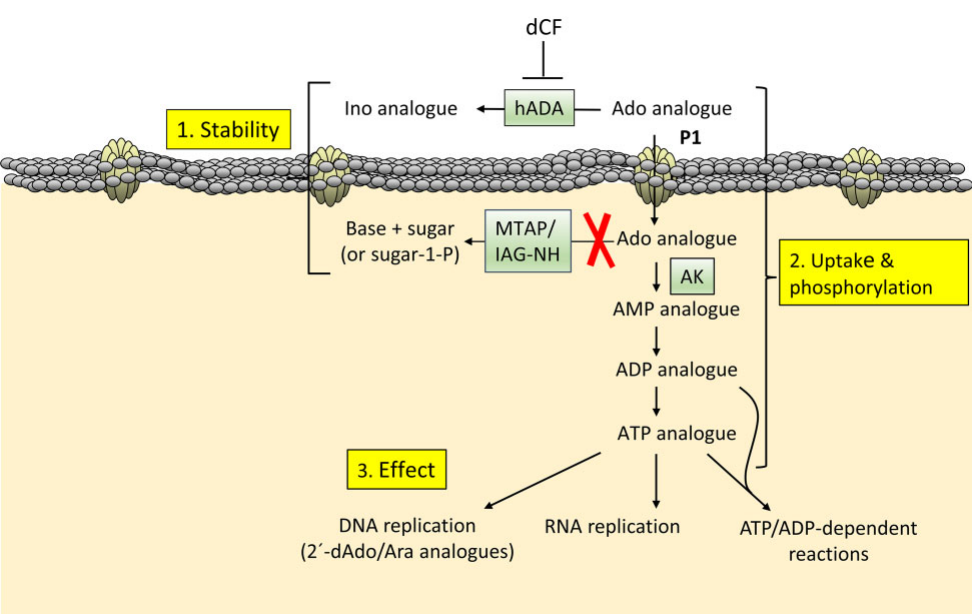
Important issues to consider when developing adenosine analogues against *T. brucei*. The scheme is valid for adenosine analogues active in their di/triphosphate form. (1) The analogue should be stable against deamination in the blood and against cleavage intracellularly. (2) The drug should be taken up predominantly by the P1 transporter and be converted to the di- or triphosphate form by kinases. (3) The active form of the drug must have an effect on cellular processes such as nucleic acid biosynthesis or ATP/ADP-dependent reactions. Abbreviations: hADA, human adenosine deaminase; dCF, deoxycoformycin; MTAP, methylthioadenosine phosphorylase; IAG-NH, inosine-adenosine-guanosine-nucleoside hydrolase; AK, adenosine kinase.

Adenosine analogues did not initially have any advantages over inosine analogues against *T. brucei* in animal models due to the fact that they are deaminated by mammalian adenosine deaminase present in the blood and are converted to the corresponding inosine analogues (Fig. 3). This situation changed when the analogues were combined with deoxycoformycin, an adenosine deaminase inhibitor. When this inhibitor was combined with a previously known antitrypanosomal adenosine analogue, cordycepin (3´-deoxyadenosine), it became possible to cure mice with *T. brucei* brain infections (Rottenberg et al. 2005). Although efficient, cordycepin has the disadvantage of predominantly being taken up by the P2 nucleoside transporter, which is associated with drug resistance when downregulated or deleted (Matovu et al. 2003, Geiser et al. 2005). The P2 transporter is comparably easy to lose because it is encoded by a single gene. For comparison, there are two adenosine kinase genes and multiple copies of the P1 transporter. Accordingly, the selection for cordycepin-resistant trypanosomes led to mutations in the *Tb*AT1 gene (P2 transporter), whereas P1 transporters, adenosine kinase, or the other genes studied were not affected (Vodnala et al. 2009). It is therefore advisable to develop adenosine analogues that are either taken up by P1 or by both transporters but to avoid analogues that are predominantly taken up by P2.

The hunt for nucleoside analogues with better P1-P2 uptake profiles led to the discovery of 2´-deoxy-2´-fluoro-arabinofuranosyl adenine (FANA-A) (Ranjbarian et al. 2017). The study of this analogue demonstrated that the selectivity of P1 over P2 cannot be fully based on competition experiments, which is the most common way to test transporter selectivity. Although competing strongly with the P2 transporter, it turned out that P1 was the major transporter as verified by knockout cells lacking the P2 transporter, which were as sensitive to the drug as the parent strain and knockout cells with reintroduced transporter. This indicated that the drug was an inhibitor of P2 rather than a competing substrate. The FANA-A study also shed light onto the other criteria that should be fulfilled for efficient adenosine analogues against *T. brucei*. The criteria are summarized in Fig. 3 and are valid for analogues active in their nucleotide form, which is the case for most of the studied adenosine analogues. First of all, the analogues need to be stable against both deamination in the blood and to cleavage inside the trypanosomes. IAG-NH is generally considered the main cleavage enzyme in *T. brucei*, but it is rather specific for the natural purine ribonucleosides (Parkin 1996). It is therefore more important to be aware of MTAP, which has a relatively broad sugar moiety specificity and cleaves adenosine, deoxyadenosine, and to a lesser extent cordycepin (Vodnala et al. 2016). In the case of FANA-A, the 2´-fluorinated arabinosyl moiety made it resistant to cleavage (Ranjbarian et al. 2017). In addition to the stability and uptake efficiency by the P1 transporter, the analogues should also be efficiently phosphorylated in order to have a final effect on the trypanosomes. In the case of FANA-A, the triphosphate form of the drug is incorporated into DNA and blocks further replication, whereas ribonucleoside analogues block RNA synthesis and/or interfere with catabolic, anabolic, or regulatory processes by mimicking ADP or ATP. An example of the latter category is tubercidin, which in its triphosphate form inhibits 3-phosphoglycerate kinase during glycolysis (Drew et al. 2003). The selectivity of the analogues is thus based on a combination of the stability, uptake, phosphorylation, and sensitivity of the final target in the trypanosomes compared to mammalian cells.

**Figure 4.**
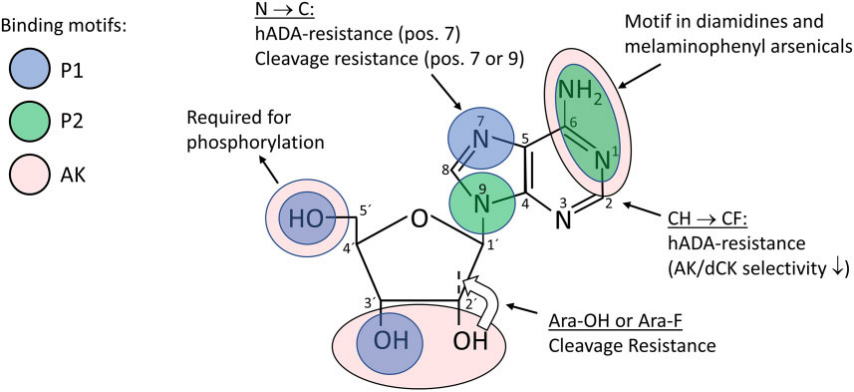
Guide for the development of adenosine analogues against *T. brucei*. The figure shows the role of different parts of the adenosine molecule on uptake, phosphorylation, cleavage, and deamination. The color codes indicate recognition motifs for the P1 and P2 transporters as well as for adenosine kinase. The white arrow indicates the swapped orientation of the OH and H groups on the 2´ carbon for arabinoside-based adenosine analogues. Abbreviations: hADA, human adenosine deaminase; AK, adenosine kinase; dCK, deoxycytidine kinase.

The establishment of the factors presented in Fig. 3 made further drug development more rational, especially concerning the role of MTAP, which has been neglected in the past. Figure 4 summarizes the influence of different positions of the adenosine molecule for stability against deamination and cleavage, P1/P2 transport, and phosphorylation by adenosine kinase. Neither cordycepin nor FANA-A are stable against deamination and needed to be combined with deoxycoformycin (pentostatin) to prevent deamination in the blood (Rottenberg et al. 2005, Ranjbarian et al. 2017). Although deoxycoformycin is a commonly used drug against some leukemias, experiments in rodents showed that it can cause birth defects and it is therefore not recommended during pregnancy (Dostal et al. 1991). Thus, it is desirable to avoid deoxycoformycin and instead use the analogues as single agents. One known way to prevent deamination is by 2-halogenization of the nucleobase moiety, but 2-fluorination makes the analogues poorer substrates of *T. brucei* adenosine kinase (Vodnala et al. 2008, Ranjbarian et al. 2017). Another drawback is that this modification will make 2´-deoxyadenosine analogues such as FANA-A (but not cordycepin) more efficiently phosphorylated by mammalian deoxycytidine kinase and thereby increase the risk of side effects (Arnér and Eriksson 1995). Experiments with 2F-cordycepin showed that it could cure *T. brucei*-infected mice as a single agent, although it required a 15-fold higher dose than cordycepin itself and has not yet been verified in a second-stage disease model (Vodnala et al. 2013).

The studies of cordycepin and FANA-A paved the way for the development of cordycepin-tubercidin hybrid analogues (*i.e*. 3´-deoxy-7-deazaadenosine analogues) based on the knowledge from previous studies that tubercidin is resistant to *T. brucei* MTAP cleavage (Vodnala et al. 2016) and to deamination (Adamson et al. 1977). It was found that 3´-deoxy-7-deazaadenosine could cure second-stage disease, and its high stability made it possible to use orally as a single agent (Hulpia et al. 2019). Analogues with varying P1/P2 specificity were identified, but it was only 3´-deoxy-7-deazaadenosine that did not show obvious side effects in the mice, and this analogue was mainly taken up by the P2 transporter. The challenge for future drug development is to improve the P1 specificity, and Fig. 4 gives some hints as to what can be done. The nucleobase recognition patterns of the P2 transporter and adenosine kinase are to a large extent overlapping, whereas the recognition pattern of P1 (as well as the sugar recognition pattern of adenosine kinase) is often altered in the analogues, with cordycepin lacking one P1-binding site (3´-OH) and 3´-deoxy-7-deazaadenosine lacking two P1-binding sites (3´-OH and N-7). To maintain a reasonable affinity for the P1 transporter, it is therefore important to add compensatory modifications to regain the lost binding strength. In the case of 7-deazaadenosine analogues, this can be done by adding substituents to position 7 of the purine ring, and verification in P2 knockout trypanosomes showed comparable drug sensitivities as the parent strain to these analogues, indicating that they are P1 specific (Hulpia et al. 2020b). Some other variants of 7-deazaadenosine analogues have also shown good activity against *T. cruzi* and *Leishmania* (Bouton et al. 2021a, Bouton et al. 2021b).

Figure 4 also shows the binding sites for *T. brucei* adenosine kinase. FANA-A and cordycepin lack a hydroxyl group at the 2´ or 3´ position, respectively, resulting in a 2–3 orders of magnitude lower affinity than adenosine. Considering that the affinity for adenosine is exceptionally high to start with (K_m_ = 41 nM), a decrease by a few orders of magnitude seems therefore to be compensated for by the active transporters that can concentrate the drugs in the cell making both drugs active with EC_50_ values in the nanomolar range against the parasite. A take-home message is that maintaining strong affinity for adenosine kinase is less critical than cleavage/deamination resistance and P1 transport efficiency when developing new analogues against *T. brucei*, and losing one interaction point with the substrate is still acceptable. A few analogues developed against *T. brucei* are also adenosine kinase-independent. These include MTAP substrate analogues that are modified at the 5´-position and thus cannot be phosphorylated at this site (Sufrin et al. 2008). MTAP has an important role in polyamine metabolism via its cleavage of methylthioadenosine, and inhibitors of the enzyme could also cure *T. brucei*-infected mice, although the therapeutic effect was dependent on the specific strain of the parasite (Sufrin et al. 2008).

## Biosynthesis of pyrimidine ribonucleotides

**Figure 5.**
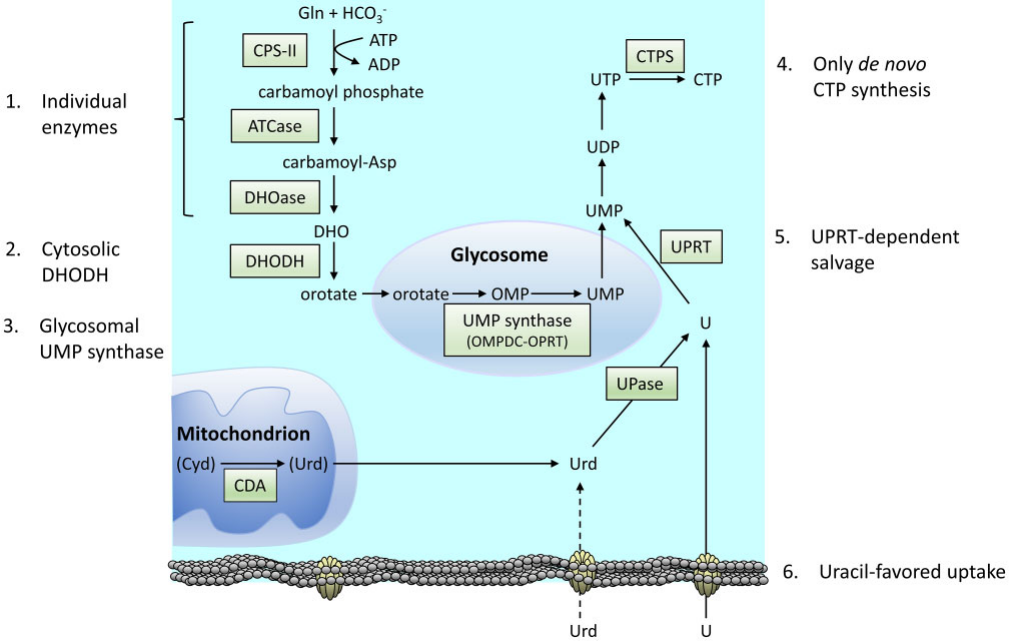
Pyrimidine metabolism in *T. brucei* with features different from mammalian cells indicated (labeled 1–6). The brackets around mitochondrial cytidine (and uridine formed downstream) indicate that cytidine is not likely to be a significant source of pyrimidines.in the parasite. Abbreviations: CPS-II, carbamoyl phosphate synthase II; ATCase, aspartate transcarbamoylase (or aspartate carbamoyltransferase); DHOase, dihydroorotase; DHOHD, dihydroorotate dehydrogenase; OMPDC, OMP decarboxylase; OPRT, orotate phosphoribosyltransferase; CDA, cytidine deaminase; UPase, uridine phosphorylase; UPRT, uracil phosphoribosyltransferase; CTPS, CTP synthetase.

Pyrimidine ribonucleotide metabolism in *T. brucei* is quite the opposite compared to that of purines by being mainly based on *de novo* synthesis instead of salvage. UTP and CTP can both be synthesized *de novo*, whereas salvage is restricted to uracil and to a lesser extent uridine (Fig. 5). A more detailed review of pyrimidine metabolism in *Trypanosomatidae* was published in 2018 (Tiwari and Dubey 2018), and here we focus on the most interesting features from the viewpoint of drug development against *T. brucei*. The pyrimidine metabolism of trypanosomatids is in many ways different from that in mammals:

- The first three enzymes are individual in the trypanosomatids (Gao et al. 1999, Tiwari and Dubey 2018), whereas they are merged into a multifunctional protein in mammals called CAD from the initials of the constituent enzymes.
- The next enzyme, dihydroorotate dehydrogenase, is cytosolic in *T. brucei* and in other trypanosomatids instead of mitochondrial as in mammals (Hammond and Gutteridge 1982, Tiwari and Dubey 2018).
- Similarly to the mammalian enzyme, the UMP synthase in *T. brucei* is bifunctional consisting of two enzymatic activities (OMPDC-OPRT in Fig. 5), but it is located to the glycosomes instead of the cytosol in the trypanosomatids (Hammond and Gutteridge 1982, Guther et al. 2014).
- In *T. brucei*, CTP can only be made *de novo*, and the intracellular levels of CTP are extremely low in long slender BSFs (Hofer et al. 2001). For comparison, mammalian cells have both *de novo* and salvage synthesis of CTP. Much less is known about the metabolism of CTP in other trypanosomatids, but cytidine/cytosine transport is limited and they generally have a CTP synthetase gene indicating that they are likely to mainly depend on *de novo* CTP synthesis as well.
- A general theme is that uridine needs to be cleaved prior to its salvage in trypanosomatids. This means that the salvage of both uracil and uridine is strictly dependent on uracil phosphoribosyltransferase (Hammond and Gutteridge 1982). This enzyme does not exist in mammals, which instead salvage uridine with uridine-cytidine kinase and use cleavage solely as a step in pyrimidine degradation. In *T. brucei* the cleavage is performed by uridine phosphorylase (UPase), which is structurally different from mammalian UPase (Larson et al. 2010).

In addition, there are many differences in the synthesis of dTTP, but that is covered in the deoxyribonucleotide metabolism section. The dTTP synthesis is dependent on a mitochondrial cytidine deaminase, which besides cytidine also uses deoxycytidine as substrate. However, it is unclear whether cytidine is an important substrate of the enzyme because the parasite lacks cytidine transporters and it is not known if significant amounts are generated by dephosphorylations of cytidine nucleotides. Cytidine is therefore in brackets in Fig.   5, and the more important deoxycytidine substrate is described together with dTTP synthesis.

Most of the listed differences are universal in the trypanosomatids. However, uridine cleavage is performed by a broad-specificity nucleoside hydrolase that also recognizes inosine in *Leishmania* instead of UPase as in *T. brucei* and *T. cruzi* (Shi et al. 1999). *Leishmania* species also have a gene encoding a putative uridine kinase, but earlier studies indicate that there is no such activity in cell extracts indicating that the gene is perhaps not active (Hammond and Gutteridge 1982). As mentioned in the transporter section, the pyrimidine specificities vary among the trypanosomatids, and *T. brucei* BSFs use the U3 transporter, which strongly favors uracil over uridine (Ali et al. 2013a). The inefficient uridine transport is indicated with a dashed arrow in Fig. 5. As indicated in the figure, uridine can also be produced from cytidine by a mitochondrial cytidine deaminase. This enzyme can also use deoxycytidine as substrate as described in the dTTP synthesis section. The reaction with cytidine is most likely of minor importance for the net production of pyrimidine nucleotides due to the lack of significant cytidine transport activities. It is therefore indicated in brackets in the figure. However, it cannot be excluded that the enzyme has a role in taking care of cytidine produced internally from the dephosphorylation of cytidine-containing nucleotides.

The many differences in pyrimidine *de novo* synthesis between *T. brucei* and mammalian cells is an advantage for developing specific inhibitors targeting the trypanosome-specific features in *de novo* UTP synthesis. One of the key questions has been whether the trypanosomes can overcome the inhibition of *de novo* synthesis by uracil/uridine salvage despite the facts that uracil is barely detectable in the blood and that uridine is a poor substrate of the U2 transporter. Two studies of knockout parasites lacking UMP synthase in *de novo* synthesis showed that the cells grew readily *in vitro* as long as there were sufficient levels of pyrimidines in the growth medium, but they had difficulties surviving and proliferating inside the mammalian host (Ali et al. 2013b, Ong et al. 2013). The parasitemia was low in one of the studies and non-detectable in the other. However, the trypanosomes could in the latter case regain virulence if they were accustomed to medium containing traces of uracil/uridine (using non-dialyzed serum in the growth medium) over 4 months before infecting the mice (Ong et al. 2013). Both studies concluded therefore that blockage of *de novo* pyrimidine synthesis is possible to circumvent, and it is therefore not a good target by itself. An alternative strategy could instead be to combine an inhibitor of *de novo* synthesis with a UPRT substrate analogue. An example of such a substrate analogue is 5-fluorouracil. Both UMP knockout studies showed that the trypanosomes became >10 times more sensitive to this drug, indicating that they compensated for the blockage of *de novo* synthesis with increased salvage (Ali et al. 2013b, Ong et al. 2013). The drug combination would thus serve the dual purpose of both blocking *de novo* synthesis and preventing compensatory upregulation of the salvage pathway.

An enigma in *T. brucei* pyrimidine metabolism is why BSFs have such low CTP levels. Only about 0.5% of the total NTP level is CTP in *T. brucei* isolated from mice, whereas the corresponding percentage in cultured procyclics is 4% (Hofer et al. 2001) and is 6% in mammalian cells (Traut 1994). In contrast to mammalian cells, which can make CTP both by *de novo* synthesis and by cytidine salvage, the trypanosomes are not able to salvage cytidine or cytosine (Hofer et al. 2001). They are therefore completely dependent on CTP synthetase, which makes it a good target for chemotherapy against the parasite. *Trypanosoma brucei* lacks uridine/cytidine kinase and can therefore not phosphorylate and thereby activate cyclopentenyl cytosine, 3-deazauridine, or other pyrimidine nucleoside analogues targeting CTP synthetase. Drug development has instead focused on glutamine analogues such as 6-diazo-5-oxo-L-norleucine (DON), α-amino-3-chloro-4,5-dihydro-5-isoxazoleacetic acid (acivicin), and 3-bromoacivicin (Hofer et al. 2001, Fijolek et al. 2007, Conti et al. 2011). Glutamine analogues have the drawbacks of causing side effects on other glutamine-dependent reactions such a mammalian *de novo* purine biosynthesis (DON) and GMP synthase (acivicin). *De novo* purine biosynthesis is the major target of DON in mammalian cells, and they can therefore be rescued by supplying hypoxanthine in the growth medium whereas the trypanosomes cannot circumvent the effect of the drug on CTP synthesis (Hofer et al. 2001). 3-Bromoacivicin was developed as an improved version of acivicin with the chlorine atom replaced by bromine resulting in higher *T. brucei* selectivity (Conti et al. 2011). However, it was not possible to cure *T. brucei*-infected mice with DON, acivicin, or 3-bromoacivicin (Hofer et al. 2001, Fijolek et al. 2007, Conti et al. 2011). In all three cases, the number of parasites decreased below the detection limit but reappeared 4–6 days after the treatment was terminated. Attempts have also been made with improved versions of acivicin by designing analogues based on the crystal structure of *T. brucei* CTP synthetase, but 3-bromoacivicin is still the most selective analogue (Tamborini et al. 2012). A possible alternative to the glutamine analogues could be to use pyrazolone-based hydrazones, which were recently discovered to strongly reduce CTP pools in the parasite indicating that CTP synthetase is a likely target (Marchetti et al. 2022).

## *De novo* synthesis of deoxyribonucleotides

**Figure 6.**
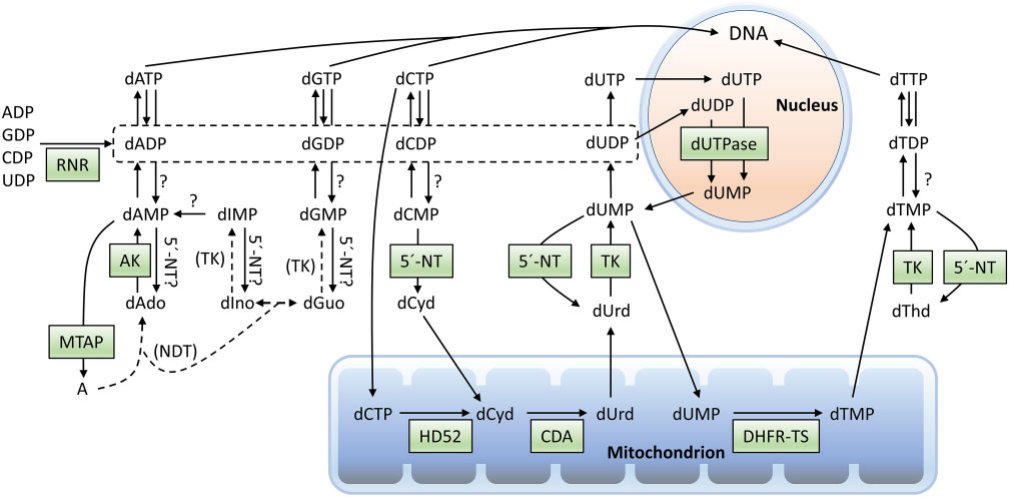
Synthesis of dNTPs in *T. brucei* takes place in the cytosol, mitochondrion, and nucleus. Note that dNTPs are needed both for nuclear and mitochondrial DNA synthesis, although only nuclear DNA synthesis is indicated in the figure. Reactions that are not studied are marked with question marks, whereas reactions supposedly of minor importance are marked with dashed arrows and associated enzymes in brackets (these are excluded from the metabolism summary in Fig. 1B). Abbreviations: RNR, ribonucleotide reductase; AK, adenosine kinase; MTAP, methylthioadenosine phosphorylase; 5´-NT, 5´-nucleotidase; TK, thymidine kinase; dUTPase, deoxyuridine triphosphate pyrophosphatase; HD52, histidine-aspartate domain-containing protein 52; CDA, cytidine deaminase; DHFR, dihydrofolate reductase; TS, thymidylate synthase; NDT, nucleoside deoxyribosyltransferase.

*Trypanosoma brucei* has a full set of enzymes to produce dNTPs by *de novo* synthesis. As shown in Fig. 6, ribonucleotide reductase (RNR) is a key enzyme for the *de novo* synthesis of all dNTPs. RNR uses NDPs as substrates and produces the corresponding dNDPs, which after a phosphorylation step by NDP kinase become the dNTPs needed for DNA synthesis. Similarly to nearly all RNRs, the *T. brucei* enzyme is subject to allosteric specificity regulation, where the binding of dATP/ATP, dTTP, or dGTP determines which of the four substrates to reduce and thereby ensures balanced levels of dNTPs in the cell (Hofer et al. 1997, Hofer et al. 1998). The binding of dATP (or ATP) to this site stimulates the reduction of CDP and UDP, with CDP being the preferred substrate of the two. This is not unique to *T. brucei*, and the mammalian RNR also has a preference for CDP over UDP. The main difference between the two RNRs is instead in the second allosteric site that normally controls the overall activity of the enzyme by binding dATP, which turns the enzyme off, and ATP that that turns it on. In *T. brucei* this site can bind the two nucleotides, but dATP is unable to switch the activity off, and it has been proposed that this function has been lost in evolution (Hofer et al. 1998). A possible benefit of losing dATP inhibition could be to enhance the synthesis of dCTP by making dATP a purely stimulatory effector for the reduction of CDP and to lesser extent UDP.

The lack of dATP inhibition has consequences for drug discovery. Many deoxyribonucleoside analogues developed against mammalian cells in cancer therapy act both by mimicking dATP to inhibit RNR via the overall activity site and by blocking DNA synthesis. It is important to be aware that these analogs do not generally affect RNR in *T. brucei*, which lacks this allosteric site, and they often act as pure DNA synthesis inhibitors instead. Some anticancer nucleoside analogues are also substrate analogues of RNR, but in this case it is important to be aware that cytidine/deoxycytidine analogues (e.g. gemcitabine) are not likely to work because *T. brucei* does not have any cytidine/deoxycytidine kinase that can phosphorylate the analogues into their active form. As a target of cancer therapy, many classes of RNR inhibitors have been developed, including free radical scavengers, iron chelators, and sulfhydryl-reacting agents. A few of them have been tested on *T. brucei*, including hydroxyurea (a radical scavenger) and thiosemicarbazones (iron chelators), but these have so far not been specific enough in comparison to mammalian reference cells (Hofer et al. 1998, Ellis et al. 2015). Nevertheless, some of the most well-known antitrypanosomal compounds act as indirect inhibitors of RNR activity by interfering with the metabolism of trypanothione, which is needed as a reductant of RNR via tryparedoxin (Dormeyer et al. 2001). Melarsoprol is an example of a sulfhydryl-reacting agent that scavenges trypanothione (and can possibly also react with sulfhydryl groups in RNR itself), whereas DFMO blocks the synthesis of trypanothione by inhibiting ornithine decarboxylase (Fairlamb et al. 1987, Fairlamb et al. 1989, Dormeyer et al. 2001, Larson et al. 2021).

The synthesis of dTTP requires several additional enzymes compared to the other dNTPs. A key enzyme in *de novo* dTTP synthesis is thymidylate synthase (TS), which converts dUMP to dTMP. The methyl group needed in the reaction is generally taken from methylene-tetrahydrofolate. In *T. brucei* and other trypanosomatids, TS is fused to dihydrofolate reductase (DHFR), which is needed for the regeneration of tetrahydrofolate from the dihydrofolate generated in the TS-catalyzed reaction (Garrett et al. 1984). Another specific feature of DHFR-TS is that in contrast to the corresponding mammalian enzymes it is mitochondrial and not cytosolic (Moro-Bulnes et al. 2019). Knockout studies in *T. brucei* showed that DHFR-TS-depleted trypanosomes require thymidine in the growth medium for survival and cannot establish infections in mice, indicating that the low thymidine levels in the blood are not sufficient for the parasite to live on (Sienkiewicz et al. 2008). Knockout trypanosomes cultured in thymidine-free medium died within 60 hours indicating that it is not only parasite proliferation that is affected, but also parasite viability.

TS and DHFR are used as targets against cancer, psoriasis (by inhibiting immune cell proliferation), and infectious diseases. Studies of the recombinantly expressed *T. brucei* DHFR-TS protein showed that typical inhibitors of mammalian DHFR (e.g. methotrexate) and TS (e.g. 5-fluoro-dUMP) were not selective enough and that microbial DHFR inhibitors such as pyrimethamine and trimethoprim were not very effective (Gibson et al. 2016). However, it is only the TS part of the protein that is essential. *T. brucei* and other trypanosomatids can compensate for inhibition of DHFR by upregulating pteridine reductase 1 (PTR1). The DHFR domain and PTR1 have been extensively studied, whereas the full-length DHFR-TS protein is much less stable and more difficult to work with (Gibson et al. 2016). PTR1 is a broad-substrate enzyme that can reduce both biopterins and folates and thereby bypass the need for DHFR. Knockout studies showed that it is essential in *T. brucei* by being the only enzyme that can reduce biopterins (Sienkiewicz et al. 2010), but not in *Leishmania* amastigotes that also have a quinonoid dihydrobiopterin reductase (QDPR) for biopterin metabolism (Ong et al. 2011). PTR1 and the DHFR domain of DHFR-TS have both been structurally determined in complex with inhibitors, and it has been possible to develop drugs targeting PTR1 alone as well as dual inhibitors affecting both enzymes with high specificity against the parasite over mammalian cells (Mpamhanga et al. 2009, Vanichtanankul et al. 2011, Khalaf et al. 2014, Pohner et al. 2022). The dual inhibitors have the advantage that they also can be used against *Leishmania* amastigotes, which have QDPR that can compensate for the inhibition of the biopterin metabolism but not the inhibition of folate metabolism (Ong et al. 2011). Only one class of dual inhibitors of DHFR and PTR1 has so far been tested in animal studies, but these inhibitors were considered toxic (Khalaf et al. 2014) and more *in vivo* data are needed to know if it is a promising approach.

It is not only the DHFR-TS protein that is unique in *T. brucei* dTTP synthesis. Perhaps even more surprising was the finding of a new pathway to produce dUMP, which is needed as a substrate of TS (Fig. 6). In most organisms, the majority of dUMP comes from the deamination of deoxycytidine nucleotides (dCMP or dCTP depending on the species). This is because RNR generally has a comparably high affinity for CDP and produces a surplus of dCDP compared to dUDP, which subsequently forms the dCMP or dCTP substrates for the deamination reaction. However, *T. brucei* lacks dCMP deaminase and dCTP deaminase, which was initially believed to be an adaption to save cytidine nucleotides that are present at very low levels in BSFs (Hofer et al. 2001). Surprisingly, it was instead found that *T. brucei* has a novel pathway for dUMP production from dCTP that is mainly mitochondrial (Moro-Bulnes et al. 2019, Yague-Capilla et al. 2021). The pathway starts with the removal of all three phosphates from dCTP by a mitochondrial orthologue of the mammalian enzyme sterile α-motif histidine-aspartate domain-containing protein 1 (SAMHD1) (Yague-Capilla et al. 2021). The mammalian SAMHD1 dephosphorylates dNTPs both as a way to limit their concentrations when they are not needed (outside S-phase) and as a defense system against DNA viruses. Knocking out the *T. brucei* orthologue, HD52, led to increased dCTP and decreased dTTP pools, and it was concluded that dCTP must be an important HD52 substrate (Yague-Capilla et al. 2021). The gene was found to be conditionally essential, and the trypanosomes could be rescued by supplying thymidine in the growth medium, indicating that the enzyme is important for dTTP synthesis (Yague-Capilla et al. 2021). The second mitochondrial enzyme in the pathway is cytidine deaminase, which converts the deoxycytidine formed in the previous step into deoxyuridine (Moro-Bulnes et al. 2019). The deoxyuridine is subsequently exported to the cytosol where thymidine kinase uses it for the synthesis of dUMP, which needs to be transported back into the mitochondrion to be used as a substrate of DHFR-TS (Moro-Bulnes et al. 2019).

It is not yet known how the dTTP synthesis pathway is regulated. In mammalian cells, dCMP deaminase is inhibited by dTTP and activated by dCTP, and it can thereby regulate the proportion of dCTP that is converted into dTTP. In *T. brucei*, DH52 is a possible candidate for the regulation of the dCTP-dTTP balance. The corresponding mammalian orthologue, SAMHD1, is an allosteric enzyme, whereas cytidine deaminase, TS, and thymidine kinase generally lack true allosteric regulation (thymidine kinase can be inhibited by dTTP, but this is only a competitive inhibition via the catalytic site). An interesting conclusion from the study of *T. brucei* dTTP synthesis is the unique role of thymidine kinase, which is involved in both *de novo* dTTP synthesis and thymidine/deoxyuridine salvage (Leija et al. 2016, Moro-Bulnes et al. 2019). The knockout/knockdown studies indicated that thymidine kinase is essential in contrast to cytidine deaminase and other enzymes in the pathway that were shown to be conditionally essential and could be rescued by supraphysiological thymidine concentrations (Leija et al. 2016). However, the conditionally essential enzymes might also be of interest for drug discovery because, as mentioned above for DHFR-TS, thymidine auxotrophs are generally noninfectious because thymidine concentrations in blood and other body fluids are not sufficient to support parasite survival, although it remains to be verified that they cannot regain infectivity via gradual adaptation to thymidine starvation. Other trypanosomatids share some of the features with *T. brucei* dTTP metabolism—including DHFR-TS and thymidine kinase—and they also lack dCMP deaminase, but it is not known if the whole pathway is conserved.

An alternative way to make dUMP is by dephosphorylating deoxyuridine nucleotides (produced by RNR) through the activity of dUTPase. The trypanosomatid enzymes belong to the dimeric class of dUTPases, which are mainly bacterial and structurally different from the trimeric class of enzymes found in other eukaryotes as well as in some bacteria (Harkiolaki et al. 2004). Dimeric dUTPases use both dUTP and dUDP as substrates, and the *T. brucei* enzyme was found to be nuclear (Castillo-Acosta et al. 2008). Knockout/knockdown studies showed that enzyme depletion led to growth arrest and increased incidences of mutations, strand breaks, and variable surface glycoprotein switching (Castillo-Acosta et al. 2008, Castillo-Acosta et al. 2012), a phenotype that was found to be linked to abasic sites caused by dUTP incorporation and subsequent base excision repair. The growth arrest could be rescued by adding high concentrations of thymidine in the growth medium. This was initially taken as evidence that the enzyme was needed for the supply of dUMP in dTTP synthesis (Castillo-Acosta et al. 2012), but this conclusion was made before the discovery of the pathway to make dTTP from dCTP, and it could instead be an effect of increased levels of dTTP competing with dUTP for binding to DNA polymerase.

## Salvage synthesis of deoxyribonucleotides

Salvage of deoxyribonucleosides is limited in *T. brucei*; there are only two enzymes able to phosphorylate deoxyribonucleosides, and only the thymidine kinase is fully dedicated for such purpose (Fig. 6). This is in contrast to mammalian cells, which have four dedicated enzymes and the ability to phosphorylate all natural deoxyribonucleosides in the cytosolic as well as mitochondrial compartments. *T. brucei* does not have any mitochondrial deoxyribonucleoside kinases. A main difference between the organisms is that *T. brucei* and other trypanosomatids have a single mitochondrion that replicates its DNA in coordination with the cell cycle, whereas mammals need to supply dNTPs for mitochondrial biogenesis and DNA repair also in resting cells with very low dNTP pools, and thus the ability to salvage deoxyribonucleosides can be an additional source of dNTPs.

One of the enzymes able to phosphorylate deoxyribonucleosides in *T. brucei* is the adenosine kinase described in the purine section, which phosphorylates both adenosine and deoxyadenosine. However, deoxyadenosine needs to be at supraphysiological concentrations to circumvent cleavage by MTAP and is not likely to be relevant for regular dATP synthesis (Vodnala et al. 2016). On the contrary, *T. brucei* grown in the presence of 1 mM deoxyadenosine expands its dATP pool to become the major nucleotide in the cell, and ATP that is used for deoxyadenosine phosphorylation drops to a third of its original level (Hofer et al. 1998, Vodnala et al. 2016). This situation is very harmful to the trypanosomes, which die within a few hours. At these high deoxyadenosine concentrations, MTAP cannot protect the trypanosomes due to that it becomes product-inhibited by the generated adenine (Vodnala et al. 2016). Although deoxyadenosine itself is not useful as a drug because of the high concentrations needed to avoid MTAP cleavage, analogues of deoxyadenosine that are resistant to the cleavage can be efficient antitrypanosomal agents (Ranjbarian et al. 2017). It was then concluded that the antiproliferative effect was due to DNA synthesis inhibition, whereas the cytotoxic effects seen at higher drug concentrations could be a result of the observed ATP depletion.

The second deoxyribonucleoside salvage enzyme in *T. brucei* is thymidine kinase, which as described above is also involved in *de novo* synthesis. This enzyme differs from most other thymidine kinases, including those from *Leishmania* and *T. cruzi*, by being a tandem enzyme with two homologous domains in *T. brucei* and four homologous domains in *T. congolense* (Ranjbarian et al. 2012). The tandem structure was also found in two unrelated protozoan parasites *Trichomonas vaginalis* and *Giardia intestinalis*, as well as in the parasitic round worm *Ascaris suum* indicating an overrepresentation in parasites (Ranjbarian et al. 2012, Krakovka et al. 2022). Studies of the *T. brucei* thymidine kinase showed that the C-terminal domain contains the enzymatic activity, while the N-terminal domain helps the active domain to achieve stronger binding to its substrate. Interestingly, the enzyme has a broader substrate specificity than other studied thymidine kinases. It uses thymidine and deoxyuridine as its main substrates, but it can also use deoxyinosine and deoxyguanosine. However, the addition of deoxyguanosine in the growth medium did not lead to expansion of the *T. brucei* dGTP pool, suggesting that its salvage is not very efficient (Hofer et al. 1998). Nevertheless, it should be remembered that the effect of a substrate analogue against the parasite that exploits thymidine kinase will also be dependent on the P1 transporter, which has a much higher affinity for the purines than thymidine (an ∼100-fold difference). A possible drug development strategy could therefore be to develop thymidine analogues with additional functional groups to increase the affinity for the P1 transporter. It would then be an advantage that the thymidine kinase has a broad substrate specificity and therefore can tolerate more changes in the substrate analogue than the mammalian enzyme. Two other factors that make the development of thymidine kinase substrate analogues interesting is that the enzyme seems to be essential (Leija et al. 2016) and that the transport is mediated by the P1 transporter family. Both factors minimize the risk of developing drug resistance. Studies of the protozoan parasite *G. intestinalis* indicate that substrate analogues of thymidine kinase can be efficient antiprotozoal agents (Krakovka et al. 2022).

Figure 6 also contains some reactions with question marks, including reactions performed by an HD-domain 5´-nucleotidase proposed to have a role in deoxynucleotide metabolism (Leija et al. 2016). The enzyme can to various extents use ribonucleoside and deoxyribonucleoside monophosphates as substrates, especially dCMP, dUMP, and dTMP. Potential but not tested substrates of HD-domain 5´-nucleotidase (dAMP, dIMP, and dGMP) are marked with question marks in the figure. A related uncertainty that is indicated in the figure is that it is not clear if significant levels of dNMPs are normally produced in the cell. Only limited amounts can be expected from nonspecific phosphatase activities, from the occasional use of dNTPs by enzymes using NTP-to-NMP conversions as an energy source, and from DNA repair. The only dNMPs that are produced as a part of a pathway are dUMP and dTMP, which are produced in the *de novo* synthesis of dTTP. Figure 6 also includes a reaction catalyzed by *T. brucei* NDT, which primarily interconverts deoxyribonucleosides but to some extent also ribonucleosides as described in the purine salvage section. The enzyme exchanges the nucleobase on 6-oxopurines with free purine nucleobases (A, G, or H) from the surroundings and can thereby establish an equilibrium between deoxyguanosine and deoxyinosine and also make deoxyadenosine from any of the two other purine deoxyribonucleosides (Del Arco et al. 2019). However, the physiological relevance of NDT from *T. brucei* and other trypanosomtids is unclear. Deoxyadenosine is the only one of the three deoxyribonucleosides that is efficiently salvaged in *T. brucei*, but it is generally cleaved by MTAP under physiological conditions and even if some additional deoxyadenosine can be generated from deoxyinosine/deoxyguanosine, it is not likely to be sufficent to overcome MTAP cleavage.

## Concluding remarks

Knowledge about nucleotide metabolism in *T. brucei* and other trypanosomatids has expanded significantly in recent years, leading to a better understanding of how the different pathways are compartmentalized and which targets are most promising to focus on for drug development. These findings can be summarized as follows:

- Purines can only be made by salvage synthesis in trypanosomatids. Some reactions are cytosolic, whereas others are glycosomal. A full set of interconversion enzymes between AMP, IMP, and GMP makes it possible for the parasites to survive on any purine source.
- Purine uptake is dependent on high-affinity active transporters in trypanosomatids. In *T. brucei*, the P2 transporter is relatively easy to downregulate and is associated with drug resistance. It is therefore preferable if purine nucleoside analogues are mainly taken up by the P1 transporter family instead.
- The most successful drug development projects have been with purine analogues. Adenosine analogues can cure *T. brucei*-infected mice, and some of them also cure brain infections. Nucleobase analogues (primarily allopurinol) have been more successful against the other trypanosomatids and allopurinol is a common treatment against feline and canine leishmaniasis.
- Most adenosine analogues are active in their triphosphate form, and in order to be effective against *T. brucei* they should be mainly taken up by the P1 transporter, be resistant to deamination and cleavage, and be efficiently phosphorylated. Figure 4 provides a guide for further development of adenosine analogues against *T. brucei*.
- UTP can be made both by *de novo* pyrimidine synthesis and via uracil/uridine salvage in trypanosomatids, but *de novo* synthesis is generally dominating. *De novo* pyrimidine biosynthesis is mainly cytosolic with the exception of UMP synthase, which is glycosomal. The multitude of differences between pyrimidine synthesis in *T. brucei* and mammals is highlighted in Fig. 5.
- CTP can only be made by *de novo* synthesis in *T. brucei*, and the CTP pools are extremely small in BSFs but not in procyclics. More research is needed to determine the purpose of having such low CTP pools in *T. brucei* BSFs.
- *Trypanosoma brucei* dTTP synthesis is dependent on an entirely new mainly mitochondrial pathway. This pathway, which has not yet been studied in the other trypanosomatids, includes a mitochondrial dCTP hydrolase (DH52), mitochondrial cytidine deaminase, cytosolic thymidine kinase, and mitochondrial DHFR-TS (Fig. 6). Other dNTPs can be made via RNR.
- CTP synthetase, RNR, and most likely thymidine kinase are the only completely essential enzymes in *T. brucei* nucleotide metabolism. They are therefore potentially good targets for chemotherapy. Especially the recent discovery that thymidine kinase seems essential came as a surprise. This is the first time a thymidine kinase has been shown to be involved in both salvage and *de novo* dTTP synthesis in any organism.
- There are many conditionally essential enzymes, which when knocked out make *T. brucei* dependent on growth medium supplements but GMP synthase is the only one of these enzymes verified to be essential for establishing infections in animal models so far but also in this case, it remains to be verified that the parasites cannot adapt to the situation by the upregulation of compensatory pathways.
- The nucleotide metabolism of *T. brucei* is to a large extent conserved in other pathogenic trypanosomatids but crucial experiments are often lacking. Consequently, it is not known if the novel mitochondrial dTTP synthesis pathway in *T. brucei* described above exists in *T. cruzi* and *Leishmania*, and even less is known about the nucleotide metabolism in *T. congolense* and *T. vivax*. More research is needed to fill in this obvious gap of knowledge.
